# Protective Role of R-spondin1, an Intestinal Stem Cell Growth Factor, against Radiation-Induced Gastrointestinal Syndrome in Mice

**DOI:** 10.1371/journal.pone.0008014

**Published:** 2009-11-24

**Authors:** Payel Bhanja, Subhrajit Saha, Rafi Kabarriti, Laibin Liu, Namita Roy-Chowdhury, Jayanta Roy-Chowdhury, Rani S. Sellers, Alan A. Alfieri, Chandan Guha

**Affiliations:** 1 Department of Radiation Oncology, Albert Einstein College of Medicine, Bronx, New York, United States of America; 2 Department of Medicine, Albert Einstein College of Medicine, Bronx, New York, United States of America; 3 Department of Genetics, Albert Einstein College of Medicine, Bronx, New York, United States of America; 4 Department of Pathology and Montefiore Medical Center, Albert Einstein College of Medicine, Bronx, New York, United States of America; Mizoram University, India

## Abstract

**Background:**

Radiation-induced gastrointestinal syndrome (RIGS) results from a combination of direct cytocidal effects on intestinal crypt and endothelial cells and subsequent loss of the mucosal barrier, resulting in electrolyte imbalance, diarrhea, weight loss, infection and mortality. Because R-spondin1 (Rspo1) acts as a mitogenic factor for intestinal stem cells, we hypothesized that systemic administration of Rspo1 would amplify the intestinal crypt cells and accelerate the regeneration of the irradiated intestine, thereby, ameliorating RIGS.

**Methods and Findings:**

Male C57Bl/6 mice received recombinant adenovirus expressing human R-spondin1 (AdRspo1) or E.coli Lacz (AdLacz), 1–3 days before whole body irradiation (WBI) or abdominal irradiation (AIR). Post-irradiation survival was assessed by Kaplan Meier analysis. RIGS was assessed by histological examination of intestine after hematoxilin and eosin staining, immunohistochemical staining of BrdU incorporation, Lgr5 and β-catenin expression and TUNEL staining. The xylose absorption test (XAT) was performed to evaluate the functional integrity of the intestinal mucosal barrier. In order to examine the effect of R-spondin1 on tumor growth, AdRspo1 and AdLacZ was administered in the animals having palpable tumor and then exposed to AIR. There was a significant increase in survival in AdRspo1 cohorts compared to AdLacZ (p<0.003) controls, following WBI (10.4 Gy). Significant delay in tumor growth was observed after AIR in both cohorts AdRspo1 and AdLacZ but AdRspo1 treated animals showed improved survival compared to AdLacZ. Histological analysis and XAT demonstrated significant structural and functional regeneration of the intestine in irradiated animals following AdRspo1 treatment. Immunohistochemical analysis demonstrated an increase in Lgr5+ve crypt cells and the translocation of β-catenin from the cytosol to nucleus and upregulation of β-catenin target genes in AdRspo1-treated mice, as compared to AdLacz-treated mice.

**Conclusion:**

Rspo1 promoted radioprotection against RIGS and improved survival of mice exposed to WBI. The mechanism was likely related to induction of the Wnt-β-catenin pathway and promotion of intestinal stem cell regeneration. Rspo1 has protective effect only on normal intestinal tissue but not in tumors after AIR and thereby may increase the therapeutic ratio of chemoradiation therapy in patients undergoing abdominal irradiation for GI malignancies.

## Introduction

Normal homeostasis of intestinal epithelium is maintained by an intricate cell replacement process in which terminally differentiated epithelial cells are continuously and rapidly replaced by replication and differentiation of epithelial cells (transit cells) located within the intestinal crypts. Radiation-induced gastrointestinal syndrome (RIGS) is due in part to the killing of clonogenic crypt cells with eventual depopulation of the intestinal villi [1], [2]. Crypt epithelial cells proliferate rapidly and are highly sensitive to cytotoxic agents and irradiation. Loss of this regenerating population of clonogenic cells following irradiation prevents the normal reepithelialization of the intestinal villi. This impairment leads to varying degrees of villous blunting and fusion, with attenuation and hypertrophy of the villous epithelial cells [3]. These changes result in the acute RIGS presenting with malabsorption, electrolyte imbalance, diarrhea, weight loss and potentially death. The late side effects and the sequelae of severe acute intestinal radiation injury include varying degrees of intestinal inflammation, mucosal thickening, collagen deposition, and fibrosis, as well as impairment of mucosal and motor functions [4], [5], [6]

The putative multipotent, intestinal stem cell is thought to be located at the base of the crypt, either at fourth or fifth cell position from the base [7] or as crypt base columnar cells interspersed between Paneth cells [8]. In the normal state, these cells rarely proliferate unless there is a pressure for increased production of the clonogenic self-renewing progenitor cells, which undergo rapid clonal expansion, followed by differentiation into the mature cells lining the villi. The daughter cells migrate either toward the villus differentiating into enterocytes, goblet cells, and enteroendocrine cells, that are eventually shed into the gut, or inwards to the crypt bases giving rise to Paneth cells [9]. Thus, the multipotent cells are fundamental to the maintenance of the cell population of the intestinal epithelium and it's regeneration after injury [10]. Following exposure to ionizing radiation, cells located at the base of the crypt undergo rapid apoptosis, or stop dividing temporarily or permanently. The extent of cell loss and intestinal injury is dependent on the radiation dose [11]. Therefore, the fate of the crypt after injury is determined by replacement of the clonogenic proliferating crypt cells by intestinal stem cell. If all crypt cells die, the crypt is “sterilized” and disappears within 48 hours. However, if one or more ‘clonogenic cell’ survives the insult, it rapidly proliferates regenerating the crypt within 72–96 hours with subsequent reconstitutions of the villi. Survival of the animal depends on the balance between crypt depopulation, and the efficiency and number of the surviving clonogenic cells regenerating the crypts.

The β-catenin/T cell factor (TCF) signal transduction pathway plays a critical role in the regulation of proliferation and differentiation of the intestinal epithelial cells during the regeneration and maturation process along the crypt-villus axis [12], [13]. Wnt signaling and the activation of β-catenin are important in the proliferation of the pluripotent stem cell that gives rise to crypt epithelial progenitors. The amount of Wnt proteins within the intestinal epithelial cells decreases with progression up the villus. As Wnt signaling decreases, β-catenin forms a complex with APC and axin (destruction complex), leading to the degradation of β-catenin [14]. Thus Wnt signaling is likely important to the maintenance of the undifferentiated state of intestinal crypt progenitor cells [12], [13]. Recently, a Wnt target gene, *Lg45/Gpr49*, which encodes an orphan G protein-coupled receptor, was identified as a marker of intestinal stem cells because it marked small columnar cells at the base of the crypt interspersed between Paneth cells [15]. Elegant lineage tracing experiments demonstrated that these few Lgr5+ve cells could reconstitute a villus in an adult mouse upon induction of a cre knock-in allele. The R-spondin (roof plate-specific spondin) family of proteins is comprised of novel secreted proteins, which acts as major agonists and modulators of the Wnt-β-catenin signaling pathway [16], [17]. There are four human paralogs (*R-spondin*1–4), each containing a leading signal peptide, two cystein-rich, furin-like domains, and one thrombospondin type 1 domain. Human Rspo1, a 29 kd, 263 amino acid protein, has a specific proliferative effect on intestinal crypt cells [18]. Transgenic expression of Rspo1 in mice resulted in marked hyperplasia of intestinal crypts in both small and large intestine, resulting in abdominal distension [18]. Further experiments demonstrated that Rspo1 prevented mucositis, induced by a chemotherapeutic agent, 5-flurouracil (5-FU), in mice [18] and more recently it was further demonstrated by the same group that Rspo1 protected mice from chemotherapy or radiation-induced oral mucositis [19]. In addition, systemic administration of Rspo1 decreased inflammation and reduced the loss of body weight, diarrhea and rectal bleeding in a mouse model of dextran sulfate sodium-induced colitis [20]. Based upon these findings, we hypothesized that Rspo1 would be radioprotective against RIGS and examined whether Rspo1 was involved in the recovery of the intestine from radiation injury.

## Results

### Serum Rspo1 Levels Are Increased after WBI

**Figure 1:**
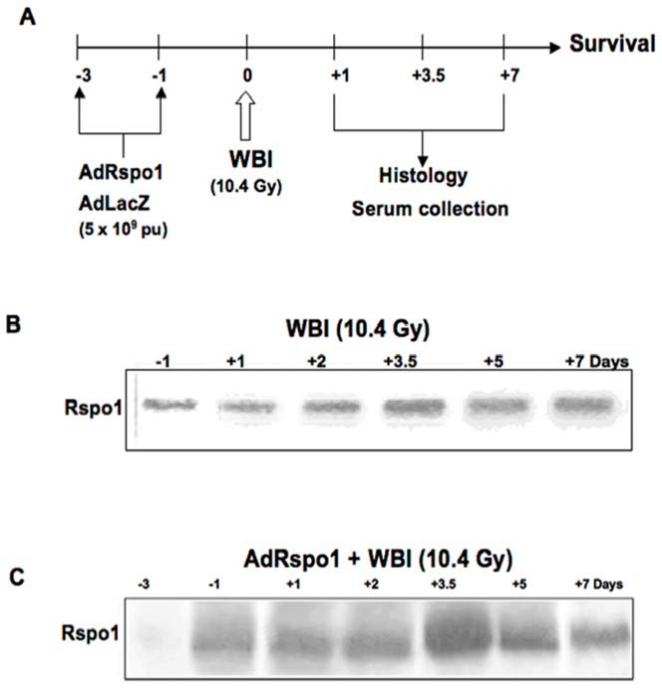
Time course evaluation of serum Rspo1 expression. (**A**) Treatment schema: AdRspo1 or AdLacZ (5×10^9^ pu) was injected intravenously 3 and 1 day before WBI (10.4 Gy) in C57Bl/6 mice. Animals were followed for survival and histological endpoints. (**B**) Immunoblots of murine serum demonstrating time course evaluation of serum Rspo1 expression after WBI. (**C**) Representative immunoblot of serum Rspo1 levels in C57Bl/6 mice, following treatment with AdRspo1 + WBI.

RIGS results in part from radiation-induced DNA damage, cell death and/or cell cycle arrest in intestinal crypt cells. Therefore, recovery from RIGS will depend on DNA repair in surviving irradiated crypt clonogens and regeneration of new intestinal progenitor cells. Since Rspo1 enhances the proliferation of intestinal crypt cells, we first examined whether the blood level of Rspo1 is increased after WBI in mice. Immunoblot analysis showed barely detectable levels of endogenous R-spondin1 in the serum of untreated mice. WBI resulted in a two-fold increase in serum Rspo1 concentrations by day 3.5 (Fig 1A and 1B). To evaluate the effect of Rspo1 on RIGS, we injected C57Bl/6J mice with 5×10^9^ particles of AdRspo1 prior to WBI (Fig 1A). Serum Rspo1 expression increased 6–8 fold in 2 to 3.5 days after AdRspo1 administration and persisted at that level for at least 1 week (Fig 1C). Mice injected with similar doses of the control adenovirus, AdLacZ showed no increase over the base line levels of Rspo1.

### AdRspo1 Improves Survival of Mice after WBI and AIR

**Figure 2:**
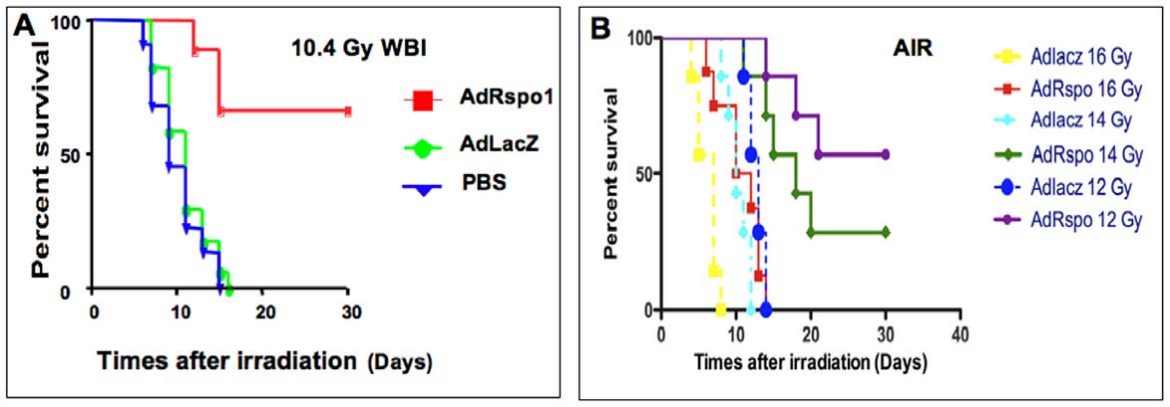
AdRspo1 treatment protected C57Bl/6 mice from radiation-induced mortality. Kaplan-Meier survival of C57Bl/6 mice treated with AdRspo1 or AdLacZ prior to WBI (10.4Gy) (Fig 2A) and 12–16 Gy AIR (Fig 2B) . Note a significant (p<0.003) increase in median survival in AdRspo1-treated mice with a median survival time of 27±1.6 days, compared to AdLacZ cohorts, 10±1.4 days. With 12–14Gy AIR median survival time for AdLacZ treated animals is 13±1.2 and 11±1.6 days compared to 25±1.3 and 19±1.4 in AdRspo1-treated animals.

In most mammals, including mice, a total-body radiation exposure of more than 10 Gy results in a characteristic gastrointestinal syndrome comprising diarrhea, weight loss and death within 5–14 days [29]. We administered escalating doses of WBI to C57Bl/6J mice to induce RIGS. Exposure to 8.4, 9.4 and 10.4 Gy was lethal in 0%, 20% and 100% of the mice within 14 days, respectively. As the 10.4 Gy dose was uniformly lethal, we administered this dose of WBI to the AdRspo1- and AdLacZ-treated groups to evaluate the radioprotective effects of Rspo1. Animals receiving WBI had diarrhea and lost body weight within 7 days. In contrast, AdRspo1-treated animals had well-formed stools and maintained body weight after WBI (23.2±0.5 g, AdRspo1 versus 17.26±1.2 g in AdLacZ-treated cohorts; p<0.0002). AdRspo1 improved survival of animals exposed to 10.4 Gy WBI significantly (p<0.003), with an improvement in median survival time from 10±1.4 days in AdLacZ treated animals to 27±1.6 days in AdRspo1-treated animals. During the first two weeks after WBI, approximately 30% of the animals died in the AdRspo1-treated group, compared with 100% mortality in AdLacZ-treated animals, indicating that Rspo1 protected these animals from RIGS (Fig 2A). The delayed mortality (after 25 days) in the AdRspo1-treated animals was interpreted to be the result of radiation-induced hematopoeitic syndrome. AdRspo1, when administered after the mice were exposed to WBI, could not mitigate the lethal effects of WBI (data not shown).

Since the effects of WBI of 10.4 Gy are secondary to combined hematopoeitic and gastrointestinal syndrome, we wanted to induce primarily a radiation-induced gastro-intestinal injury in mice. We, therefore, administered escalating doses of whole AIR after shielding the thorax, head and neck and extremities, thus protecting the bone marrow. A single fraction of 12, 14 or 16 Gy of AIR was lethal in 100% of mice treated with PBS or AdLcZ by 2 weeks. In contrast, animals treated with AIR + AdRspo1 had well-formed stools and maintained body weight (21.9±0.8, AdRspo1 versus 16.4±0.3 g in AdLacZ-treated cohorts; p<0.0001) with only 10% and 30% animals dead at 2 weeks after 12 and 14 Gy of AIR, respectively. There was significant improvement in survival in AdRspo1-treated mice to AIR doses up to 14 Gy (p<0.002) (Fig. 2B). There was no radioprotection by AdRspo1 in mice receiving 16Gy AIR.

### AdRspo1 Does Not Protect Tumors from Cytotoxic Effects of AIR

**Figure 3:**
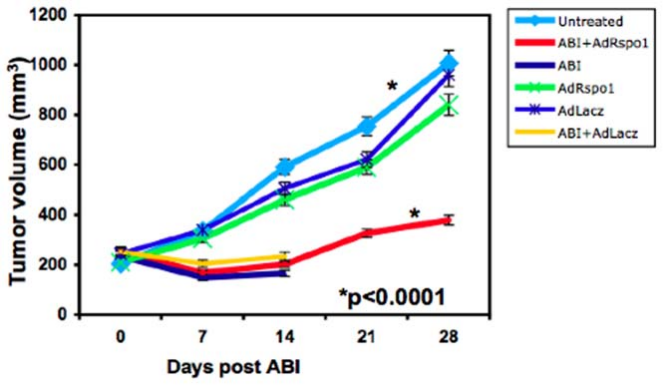
AdRspo1 treatment has no effect on tumor growth. Effect of AdRspo1 and AdLacZ treatment on tumor growth rate of Balb/c mice (n = 5) irradiated with 14Gy ABI. Significant delay in tumor growth (p<0.0001) was observed in ABI groups (Fig A) compared to untreated mice.

In order to examine whether AdRspo1 could protect tumors from radiation, Balb/c mice with palpable, murine colorectal, CT26 flank tumors were injected with either AdLacz or AdRspo1 virus, followed by 14 Gy AIR, 3 days after viral injection. AdRspo1 did not delay tumor growth compared to AdLacz. As expected, there was significant delay in tumor growth and improved survival only in AdRspo1-treated animals (median survival time 26±2 days) after AIR (Fig 3). Although, AIR reduced tumor growth (p<0.0001) but invariably produced 100% mortality of AdLacZ-treated animals. These results demonstrate that Rspo1 could increase the therapeutic ratio of radiation therapy for the treatment of abdominal tumors where it would increase the tolerance of the intestine to irradiation without providing radioprotection to the tumor.

### AdRspo1 Augments Intestinal Crypt Epithelial Cell Proliferation after WBI

**Figure 4:**
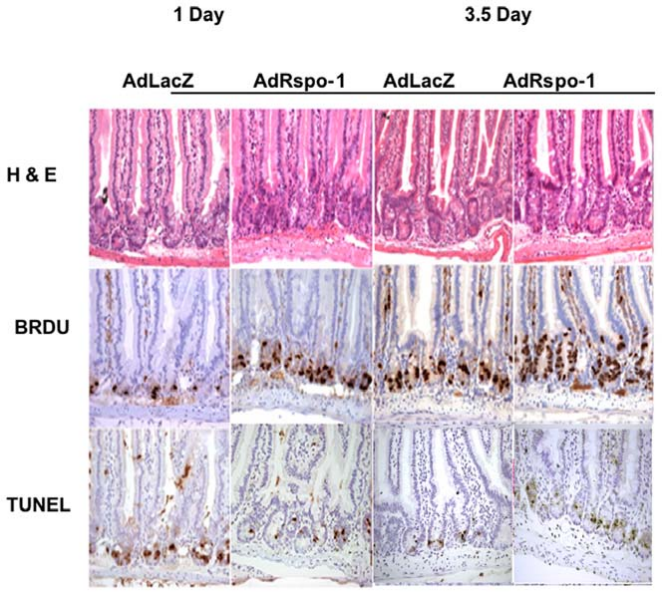
Histolological assessment of intestine after Irradiation. H&E staining demonstrates increased crypt depth and increased villi thickness in AdRspo1-treated animals following exposure to WBI. BrdU immunohistochemistry demonstrates higher crypt cell proliferation after AdRspo1 treatment when compared to AdLacZ cohorts. Finally, TUNEL staining demonstrates a decrease in the rate of TUNEL-positive, apoptotic cells in AdRspo1-treated mice post-WBI, when compared to intestinal lumen of AdLacZ-treated mice.

**Figure 5:**
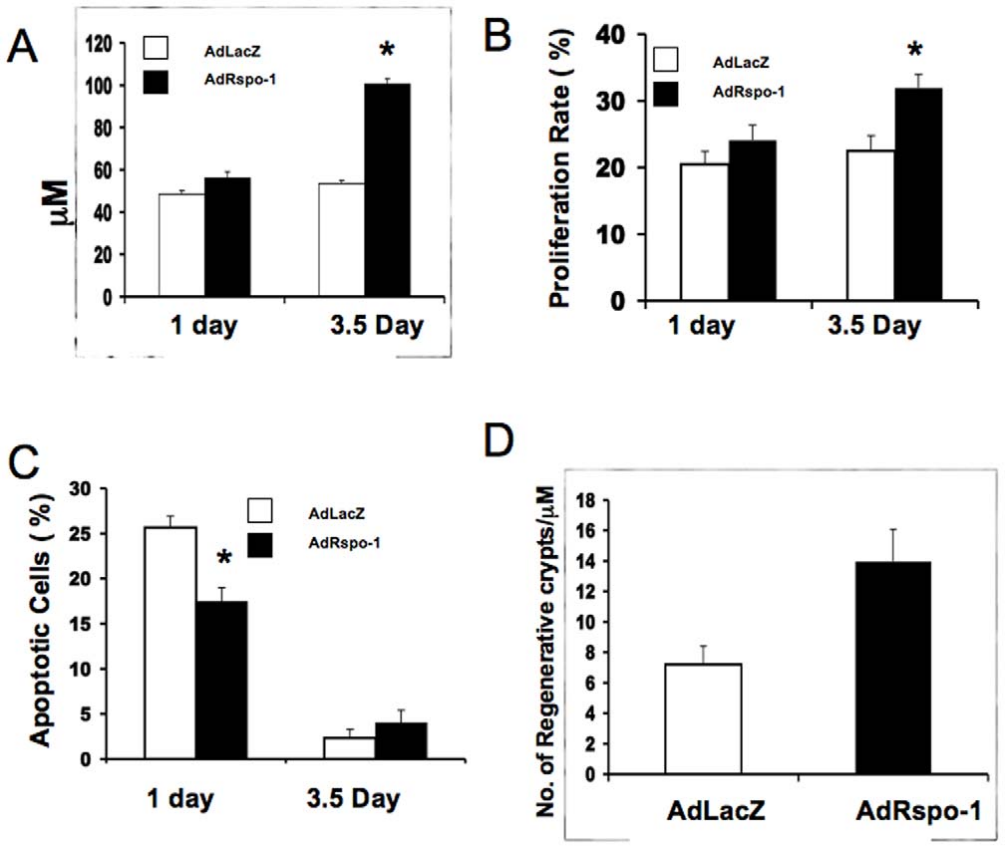
AdRspo1 increases the number of regenerative crypts in irradiated mice. Effect of AdRspo1 and AdLacZ treatment on intestinal crypt depth (A), proliferation rate (B), apoptotic cells (C) at 1day and 3.5 days after WBI and the number of regenerative crypts (D) at 3.5 days after WBI. A representative sampling of thirty crypts was assessed for each treatment group.

Radiation doses of ≥8 Gy induces cell cycle arrest and apoptosis of the crypt epithelial cells within day 1 post-radiation, leading to crypt depletion and a decrease in regenerating crypt colonies by day 3.5 and ultimately villi denudation by day 7 post-radiation exposure [23]. We, therefore, evaluated the histological manifestation of RIGS and the effect of AdRspo1 on RIGS at 1, 3.5 and 7 days, post-WBI. First, we examined whether Rspo1 induces the proliferation of crypt stem cells in mice receiving WBI. As seen in Fig 4, BrdU-labeling cells were vastly amplified in the crypts of AdRspo1+WBI-treated mice, compared to Ad-LacZ+WBI-treated controls at 1 and 3.5 days post-WBI. The percentage of the crypt epithelial cells synthesizing DNA was significantly enhanced after AdRspo1, treatment compared with those administered AdLacZ (AdRspo1, 35±2.27.versus AdLacZ, 22±2.04; P<0.05) at 3.5 days following WBI (Fig. 5B). This resulted in an increase in the overall size of the crypts, as determined by measuring crypt depth from the base of the crypt to the crypt-villus junction (Fig. 4 and 5A). A significant increase in the crypt depth in AdRspo1-treated mice compared with AdLacZ-treated mice (AdRspo1, 98.5±5.6 µm versus AdLacZ, 52±3.8 µm; p<0.001) was observed, indicating an amplification of the crypt cells after AdRspo1 treatment in irradiated mice (Fig. 4 and 5A). Finally, the intestine in WBI+AdRspo1-treated animals was much longer than those of WBI+AdLacZ-treated animals (38.48±0.9 cm AdRspo1 vs. 33.36±1.1 cm, AdLacZ; p<0.002).

### Effect of AdRspo1 on Intestinal Crypt Cell Apoptosis after Radiation Injury

Since ionizing radiation induces apoptosis of intestinal crypt epithelial cells, we performed TUNEL assay to examine apoptosis of crypt epithelial cells, 1 day after WBI. There was a significant (p<0.001) decrease in the number of apoptotic nuclei in the jejunal crypts of AdRspo1-treated animals (17±1.2) as compared with the AdLacZ-treated (26.5±1.4) controls (Fig. 4 and 5C), suggesting that Rspo1 might increase the radioresistance of the intestinal crypt compartment by decreasing radiation-induced apoptosis.

### Crypt Microcolony Assay

Radiation-induced apoptosis of crypt epithelial cells induces compensatory proliferation of intestinal stem cells and transit amplifying cells, resulting in crypt regeneration and clonal growth of damaged intestinal villi. The number of regenerating crypts forming microcolonies between days 3 and 4 after WBI, is a surrogate indicator of the resistance of the intestine to WBI and is correlated with the survival of animals from RIGS. We, therefore, counted the number of regenerative crypts per unit area of intestinal cross section, 3.5 days after exposure to WBI, according to protocols originally described by Withers and Elkind [26]. The number of crypt microcolonies was increased significantly in AdRspo1-treated mouse intestines compared with AdLacZ controls (AdRspo1, 13.8±0.7/µm versus AdLacZ, 8.2±0.5, p<0.001, Fig 5D), indicating that Rspo1 induced intestinal crypt regeneration after exposure to WBI.

### AdRspo1 Ameliorates Intestinal Malabsorption Syndrome in RIGS

**Figure 6:**
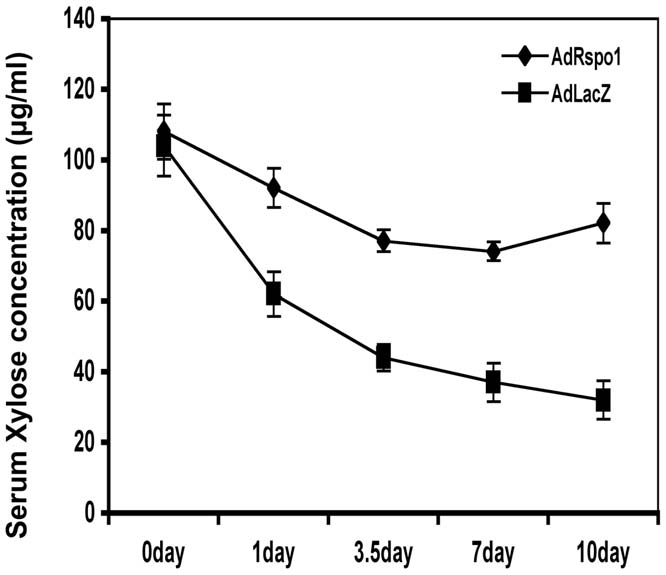
Xylose absorption assay. A time course study (1–10dys) showed significant recovery (p<0.002) of xylose absorption at 3.5 to 7 days in AdRspo1-treated cohorts, when compared to AdLacZ controls, thereby indicating the functional regeneration of intestine after radiation injury. AdLacZ-treated animals were incapable of demonstrating adequate xylose absorption after radiation injury, further contributing to animal mortality.

To evaluate the functional regeneration and absorptive capacity of the intestine, animals from various treatment cohorts were fed xylose solution following exposure to WBI. Since xylose is not metabolized in the body, serum xylose levels are a good indicator of the intestinal absorptive capacity. As expected, there was a consistent reduction in xylose absorption in AdLacZ-treated mice (33.5±7.5 g/ml), 7 days after WBI. In contrast, there was a significant recovery of xylose absorption in AdRspo1-treated mice (75±3.8 g/ml; p<0.002) at this time point. Xylose absorption continued to improve in the AdRspo-1 treated animals up to 10 days post-WBI (Fig. 6), indicating quick restitution of the intestinal villi.

### β-Catenin Localization in Nuclear and Cytosolic Fraction

**Figure 7:**
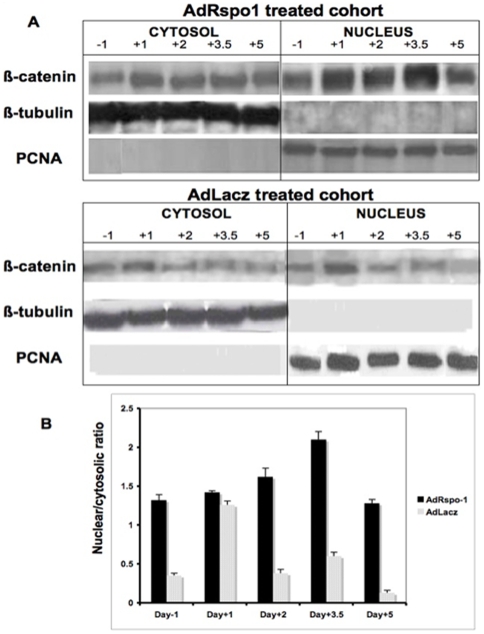
AdRspo1 treatment induces β-catenin activation in irradiated crypts. Representative immunoblot (**Fig. 7A**) and densitometric analysis (**Fig. 7B**) of nuclear/cytosolic ratios of β-catenin from AdRspo1 and AdLacZ treated cohorts after WBI(10.4Gy). Nuclear fraction purity was validated by the absence of β-tubulin, while the purity of the cytosolic fraction was evaluated by the absence of PCNA (Fig. 7A). A continuous decline in nucear/cytosolic ratios of β-catenin was predominate in samples from irradiated AdLacZ cohorts. This is further supported by the densitometric analysis of β-catenin expression (Fig. 7B) from the nuclear/cytosolic ratio demonstrating the significant differences in AdRspo1 when compared to AdLacZ treated mice prior to (Day –1) until Day +5 post WBI.

Recent reports indicate that the R-spondin proteins activate β-catenin signaling [20], [30], which is critical in maintaining intestinal homeostasis [13]. Under resting condition, β-catenin is present in the cytoplasm. Phosphorylation of β-catenin (by GSK-3 kinase) targets the proteins to to proteosomes where it is degraded. Wnt activation inhibits GSK-3 kinase phosphorylation of β-catenin, preventing β-catenin degradation and allowing for its translocation from the cytoplasm to the nucleus. In the nucleus, β-catenin binds to and activates the TCF/LEF transcription factor complex to induce the expression of wnt-pathway genes, such as, EphB2, EphB3, TCF4 and LEF1. We, therefore, examined the relative levels of β-catenin protein in the cytoplasm and nucleus of intestinal epithelial cells isolated from the two cohorts of animals that received WBI. Immunoblot analysis demonstrated a slight increase in nuclear β-catenin levels, 1 day after WBI in AdLacZ-treated mice (Fig 7A). In contrast, the nuclear/cytosolic ratio of β-catenin was much higher in Ad-Rspo1-treated mice in basal conditions (day –1, Fig 7B), which further increased by 2–4 folds the value of AdLacZ-treated animals, with a peak around 3.5 days upon exposure to WBI (Fig 7A and B). Immunohistochemistry confirmed an increase in nucelar β-catenin staining in the crypt progenitor cells in AdRspo1-treated animals, suggesting that Rspo1 enhanced stabilization and nuclear translocation of β-catenin in crypt cells in these animals (data not shown).

### AdRspo1 Amplifies the Number of Lgr5-Positive Crypt Stem Cells

**Figure 8:**
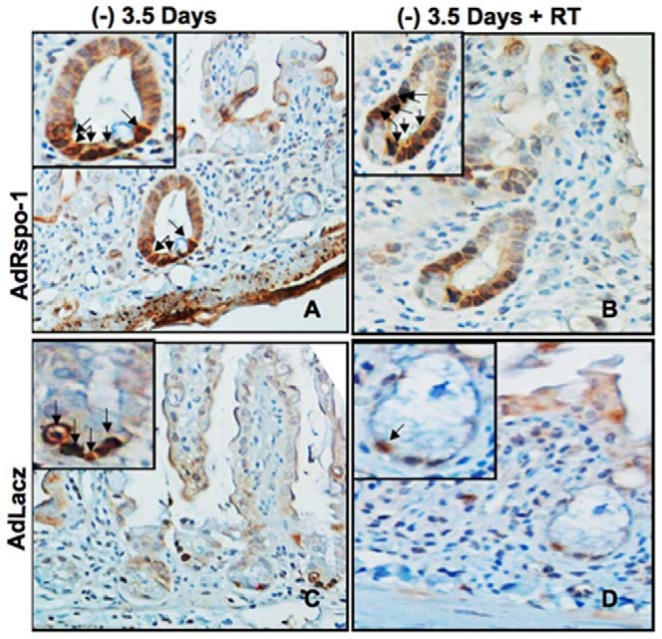
AdRspo1 treatment increases the number of Lgr5-positive intestinal stem cells in irradiated crypts. Immunohistochemical staining of Lgr5 in murine jejunum crypts at 3.5 days prior and after WBI. There was an increase in the number of Lgr5 postive cells at crypt columnar base in AdRspo1 treated cohorts when compared to AdLacZ (magnification 60x; *arrows*).

Immunohistochemical staining of murine jejunum crypts showed a significant increase in the number of Lgr5-expressing intestinal stem cells at crypt columnar base in the AdRspo1-treated mice (Fig. 8). Three and a half days after exposure to WBI, while the Lgr5+ve crypt stem cells decreased in AdLacZ-treated mice, these cells remain amplified in AdRspo1-treated mice, suggesting an expansion of the crypt stem cell compartment contributed to the protection from RIGS.

### Real Time PCR of the Expression of β-Catenin Target Genes

The expression of target genes of the β-catenin pathway in these animals was determined by realtime PCR. The mRNA levels of EphB2 and EphB3 were found to be increased by 1.85 fold and 4.8 fold, respectively in AdRspo1-treated animals exposed to WBI, as compared with AdLacZ-treated cohorts. The mRNA levels of the β-catenin target genes, TCF4 and Lef1 were also upregulated approximately 2.5 fold in response to Rspo1 after irradiation while the expression of TCF1 and TCF3 were unchanged.

## Discussion

The gastro-intestinal (GI) system is a major target for the somatic injuries associated with radiation and chemotherapy. Because of this, RIGS is an important cause of host vulnerability whether in medical therapeutics or in nuclear accidents or terrorism. Rspo1 was originally identified as a growth factor for intestinal crypt cells in a mouse transgenic model [18]. In a mouse xenograft model of human colon carcinoma, CT26, treatment with Rspo1 reduced the mucositis, diarrhea and weight loss caused by the chemotherapeutic agent, 5-flurouracil (5-FU), without affecting its antitumor effect [18]. Furthermore, systemic administration of Rspo1 decreased the histological and clinical manifestation of dextran sulfate sodium-induced colitis [20] and chemotherapy and radiation-induced oral mucositis [19] in mice. These data suggested that Rspo1 might play an important role in maintaining intestinal mucosal integrity.

Zhao et al demonstrated that prophylactic treatment with recombinant RSpo1 protein increased the mucosal thickness and reduced ulceration in the oral mucosa after irradiation and chemotherapy, presumably by increasing the proliferation of the mucosal epithelium in the basal layer of the tongue [19]. Although, Rspo1 protected radiation-induced oral mucosal injury, the effect of Rspo1 in the functional regeneration of the intestinal mucosal epithelium and amelioration of RIGS has not been studied. In this report, we demonstrate that Rspo1 is induced after exposure to WBI as a physiological response to irradiation exposure. Systemic administration of an adenovirus expressing recombinant Rspo1 amplified the Lgr5+ve intestinal crypt stem cell population and ameliorated RIGS and improved survival of mice. The effect of AdRspo1 on the regeneration of the intestinal mucosa after irradiation was manifested physically by significantly higher intestinal length and diameter, increased crypt depth and proliferative index, decreased crypt epithelial apoptosis, increased regenerative crypt microcolonies and maintenance of the villi length. This improved clinical, gross, and histopathological effects on the small intestine after WBI and AIR in AdRspo1-treated mice were physiologically manifested by a marked and progressive restoration of the normal absorptive function of the intestine, as measured by xylose absorption test.

R-spondins are a family of secreted proteins that are expressed in the small intestine, kidney, prostate, adrenal gland and pancreas [18] and are potent activators of the Wnt-β-catenin pathway [31]. Rspo1 has been demonstrated to bind with high affinity to the Wnt co-receptor, LRP6, to induce phosphorylation, stabilization and nuclear translocation of cytosolic β-catenin, thereby activating TCF/β-catenin-dependent transcriptional responses in intestinal crypt cells [32]. Our results suggest that the induction of Rspo1 after TBI may be an important protective pathway in the repair of intestinal injury in RIGS. In our experiments, Rspo1 could not prevent the mortality of the animals from the hematopoeitic syndrome, since all animals receiving WBI + AdRSpo1 were dead by 25–28 days. However, Rspo1 protected the death from GI syndrome, even with higher doses of AIR (12–14 Gy). Rspo1 likely promotes protection of RIGS through a combination of reduced radiation-induced apoptosis (i.e. increased cell survival), increased crypt cell proliferation with enhanced crypt regeneration, and rapid restoration of the structure and absorptive function of the villi. On a cellular level, AdRspo1 treatment increased the levels of nuclear β-catenin and wnt target gene expression in irradiated crypt cells. Notable among the wnt target genes that are induced in AdRspo1-treated animals are Tcf4 and Lef1, two genes that are responsible for intestinal epithelial cell proliferation and maintenance of homeostasis. Similarly, EphB2 and EphB3 are induced and could mediate crypt cell proliferation, differentiation and cell positioning along the crypt villus axis, following WBI. Furthermore, the number of Lgr5+ve crypt base columnar cells, resembling the intestinal stem cells as described by Cheng and Leblond [8], was amplified in AdRspo1+WBI-treated mice. These data, in conjunction with the histological findings of an increase in crypt regeneration and improved intestinal restitution after WBI in mice treated with AdRspo1, as compared to AdLacZ, indicates that Rspo1 mediates induction of an intestinal regenerative process, possibly as a salvage mechanism, following exposure to WBI. Furthermore, compared with AdLacZ-treated controls, pretreatment with AdRspo1 reduced WBI-associated intestinal crypt cell apoptosis. Since the wnt/β-catenin signaling has been postulated to promote radioresistance of mammary epithelial stem cells [33], Rspo1 might also confer radioprotection to crypt progenitor cells by stimulating Wnt-β-catenin signaling in RIGS.

Several growth factors and cytokines including KGF, TGFbeta, TNFα, PGE2, IL11 [34], [35], [36], [37] have been shown to protect intestine from radiation or other cytotoxic injury by increasing the crypt cell proliferation and survival. While growth factors, such as, bFGF could minimize the radiation induced intestinal damage by reducing apoptosis [38], [39]. To our knowledge, this is the first demonstration of the salutary effect of Rspo1 in the context of radiation injury of the intestine where it played a protective role by amplifying the stem cell population along with inhibition of radiation induced apoptosis in crypt. Since, Rspo1 has no protective effect on tumors during chemotherapy [18] and radiation therapy (Fig 3), systemic use of Rspo1, by protecting the normal intestinal tissue, may increase the therapeutic ratio of chemoradiation therapy in patients undergoing abdominal irradiation for GI malignancies. While the mechanism(s) associated with preserving structural regeneration and function ensures the potential prophylactic and salvage role of hRspo1 in rescuing the absorptive capacity of intestine, further studies are warranted to evaluate its potential as a therapy for RIGS in combination with other mitigating agents by reversing radiation-induced injury of the intestine.

## Materials and Methods

### Animals

Five- to 6-weeks-old male C57Bl/6 mice (NCI-Fort Dietrich, MD) were maintained in the animal maintenance facilities and all animal studies were performed under the guidelines and protocols of the Institutional Animal Care and Use Committee of the Albert Einstein College of Medicine.

### Adenovirus Construction and Administration

Since recombinant Rspo1 was not available to us, we constructed an adenovirus (AdRspo1) expressing human R-spondin1 protein and used adenoviral gene transfer for proof-of-concept experiments. Human R-spondin1 cDNA (Origene, Rockville, MD) was subcloned in pShuttle-2 (Clonetech, Mountain View, CA), followed by ligation into the Adeno-X viral DNA according to protocols described in the Adeno-XTM expression system (Clonetech, Mountain View, CA). The recombinant adenoviral vector was linearized with Pac-1 and transfected in 293 kidney cells (ATCC, Manassas, VA) using Lipofectamine plus (Invitrogen, Carlsbad, CA), according to the manufacturer's protocol until a cytopathetic effect (CPE) appeared. 293 cells were cultured in Dulbecco's Modified Eagle's medium (DMEM) (Invitrogen, Carlsbad, CA) supplemented with 10% heat-inactivated fetal bovine serum (FBS) (Atlanta Biologicals, Lawrenceville, Ga), and supplemented with 0.1 mM nonessential amino acids (Invitrogen, Carlsbad, CA), 100 µg/ml streptomycin (Invitrogen, Carlsbad, CA) and 100 units/ml penicillin (Invitrogen, Carlsbad, CA). Viral lysates were amplified and subjected to CsCl_2_ gradient centrifugation to purify the recombinant AdRspo1 adenovirus as described[21]. The adenovirus expressing the ß-galactosidase gene of E. coli (AdLacZ) was used as a control adenovirus in these experiments. All viruses were stored as 5×10^10^ particles/ml of glycerol buffer.

5×10^9^ particles of AdRspo1 or AdLacZ (adenovirus expressing *β-galactosidase* gene of E. coli as control) were injected intravenously via tail vein, 1–2 times at 3 and/or 1 day before whole body irradiation (WBI). Viral lysates were amplified and subjected to CsCl_2_ gradient centrifugation to purify the recombinant AdRspo1 adenovirus as described elsewhere [21], [22]. All viruses were stored as 5×10^10^ particles/ml of glycerol buffer.

### Irradiation Procedure

Whole-body irradiation (WBI) was performed on anesthetized mice (intraperitoneal ketamine and xylazine 7∶1 mg/ml for 100 l/mouse) using a Shephard^137^Cs -ray irradiator at a dose rate of 236cGy/min following biosafety guidelines of Albert Einstein College of Medicine. Initially a dose response (8–10.4 Gy) of WBI demonstrated that C57Bl/6 mice receiving 10.4 Gy died within two weeks, suggesting death from RIGS. Thereafter, protection experiments with AdRspo1 were performed with 10.4 Gy. Since 10.4 Gy WBI can induce both hematopoeitic and gastrointestinal injury, we also administered escalating doses (12–16 Gy) of whole abdominal irradiation (AIR) after shielding the thorax, head and neck and extremities and protecting a significant portion of the bone marrow, thus inducing predominantly RIGS.

### Irradiation of Abdominal Tumors

Balb/c mice were injected with 1×10^6^ CT26 colon cancer cells (ATCC, Manassas, VA) on the flank. Ten days after tumor inoculation, animals with palpable tumors received an intravenous injection of AdRspo1 (1×10^11^ particles), followed by whole AIR of 14Gy by Mark I^137^ Cs source a day later.

### Detection of Rspo1 Expression in Blood

Blood was drawn from the retro-orbital plexus and serum was isolated by centrifugation at 10,000 rpm for 5 min. Serum protein concentration was determined by Bradford assay kit (Bio-Rad Laboratories, Hercules, CA). Approximately 100 µg of protein was subjected to 14% SDS-PAGE, followed by electroblotting onto polyvinylidene difluoride membranes. The blot was blocked with 5% skim milk in Tris-buffered saline (10 mM Tris-HCl (pH 7.4), 150 mM NaCl, 0.05% Tween 20) followed by incubation with primary antibody (1∶200 dilution), goat polyclonal anti mouse Rspo1 (R & D Systems, Minneapolis, MN), and then with secondary antibody (1∶500 dilution), horseradish peroxidase (HRP) conjugated bovine anti-goat antibody (Santa-Cruz Biotechnology, Inc., Santa Cruz, CA). The blots were developed using Enhanced Chemiluminence assay (Amersham Pharmacia Biotech, Inc, Piscataway, NJ).

### Histology

Since radiation doses greater than 8 Gy induces cell cycle arrest and apoptosis of the crypt epithelial cells within day 1 post-radiation, resulting in a decrease in regenerating crypt colonies by day 3.5 and ultimately villi denudation by day 7 post-radiation exposure [23], we sacrificed animals when moribund or at 1, 3.5 and 7 days after WBI or AIR for time course experiments and intestine were harvested for histology. The intestine of each animal was dissected, washed in PBS to remove intestinal contents and the jejunum was fixed in 10% neutral buffered formalin prior to paraffin embedding. Tissue was routinely processed and cut into 5 µm sections for hematoxylin and eosin and immunohistochemical staining. All haemotoxylin and eosin (Fisher Scientific, Pittsburgh, PA) staining was performed at the Histology and Comparative Pathology Facility in the Albert Einstein Cancer Center. A total of 30 crypts were examined per animal for all histological parameters.

### Crypt Proliferation Rate

To visualize villous cell proliferation, each mouse was injected intraperitoneally with 120 mg/kg BrdU (Sigma-Aldrich, USA) 2–4 hrs prior to sacrifice and mid-jejunum was harvested for paraffin embedding and BrdU immunohistochemistry. Tissue sections were routinely deparaffinized and rehydrated through graded alcohols and incubated overnight at room temperature with a biotinylated monoclonal BrdU antibody (Zymed, South Francisco, CA). Nuclear staining was visualized using Streptavidin-peroxidase and diaminobenzidine (DAB) and samples were lightly counterstained with hematoxylin. Jejunum from mice, not injected with BrdU, was used as a negative control. Murine crypts were identified histologically according to the criteria established by Potten et al [24]. Digital photographs of crypts were taken at high (400–600X) magnification (Zeiss AxioHOME microscope) and crypt epithelial cells (paneth and non-paneth) intestinal sections were examined using ImageJ software and classified as BrdU positive if they grossly demonstrated brown-stained nuclei from DAB staining or as BrdU negative if they were blue stained nuclei. The proliferation rate was calculated as the percentage of BrdU positive cells over the total number of cells in each crypt.

### Determination of Crypt Depth

Crypt depth was independently and objectively analyzed and quantitated in a blind fashion from coded digital photographs of crypts from H&E stained slides using ImageJ 1.37 software to measure the height in pixels from the bottom of the crypt to the crypt-villus junction. This measurement in pixels was converted to length (in µm) by dividing with the following a conversion factor (1.46 pixels/µm).

### Detection of Apoptosis In Situ

Apoptotic cells were detected *in situ* by performing TUNEL (TdT–mediated digoxigenin labeled dUTP nick end labeling) staining. Briefly, paraffin embedded sections were de-paraffinized, rehydrated through graded alcohols and stained using an ApopTag kit (Intregen Co, Norcross, Georgia). The apoptotic rate in crypt cells was quantified by counting the percent of apoptotic cells in each crypt with analysis restricted to “intact” longitudinal crypt sections in which the base of the crypt was aligned with all the other crypt bases and the lumen [3], [24].

### In Vivo Crypt Microcolony Survival Assay

Intestinal crypt survival was measured using a modification of microcolony assay [25], [26]. A regenerative crypt comprised of tightly compacted and occasionally multi-layered large epithelial cells with a highly basophilic cytoplasm and large nuclei. The viability of each surviving crypt was confirmed by immunohistochemical detection of BrdU incorporation into five or more epithelial cells within each regenerative crypt. A minimum of four complete cross-sections was scored for each mouse and representative kinetic data were obtained from two mice in each group. Because the size of the regenerating crypt may not be the same for each treatment group, the number of surviving crypt per cross section was normalized to crypt size. Surviving crypts were defined as containing 10 or more adjacent chromophilic non-Paneth cells, a Paneth cell and lumen [25].

### Immunohistochemistry

For immunohistochemical staining of formalin-fixed, paraffin-embedded tissue sections, endogenous peroxidase activity was blocked for 30 min with methanol containing 0.3% H_2_O_2_. Antigen retrieval was performed by heating slides in pH 6.0 citrate buffer at 100°C for 20 min in a microwave oven at 500 watts. Non-specific antibody binding was blocked for 20 minutes by incubation with 10% normal rabbit serum. Sections were incubated with primary monoclonal antibody against β-catenin diluted 1∶200, and Lgr5 diluted 1∶250 (Transduction Laboratories, Lexington, KY), either 1 hr at room temperature or overnight at 4°C. The primary antibody was visualized using a streptavidin-biotin-peroxidase (ABC) kit (DAKO, Carpinteria, CA) with diaminobenzidine tetrahydrochloride (3,3′-diaminobenzidine) as the chromogen. These sections were then lightly couterstained by haematoxylin (Fisher Scientific, Pittsburg, PA).

### Isolation of Intestinal Epithelial Cells

Intestinal epithelial cells were prepared from the jejunum of adult male C57Bl6 mice by modification of the protocol described by Weiser and Ferraris [27]. Briefly, mice were anaesthetized and a catheter was inserted into the intestine through an incision in the most proximal part of duodenum. A second incision was made just proximal to the cecum and the entire small intestine was perfused with ice-cold PBS and then flushed twice with ice-cold PBS plus 1 mM dithiothreitol (DTT). The duodenum and ileum were discarded and the entire jejunum was tied at the distal end and filled to distension with isolation citrate buffer (0.9% NaCl, 1.5 mM KCl, 27.0 mM Na Citrate, 8.0 mM KH_2_PO_4_ and 5.6 mM Na_2_HPO_4_, pH 7.3) heated to 37°C for 15 mins. After incubation, the jejunum was emptied and filled with 5 ml ethylene diamine tetra acetic acid (EDTA) buffer (0.9% NaCl, 8 mM KH_2_PO_4_, 5.6 mM Na_2_HPO_4_, 1.5 mM Na_2_-EDTA, pH 7.6, plus 0.5 mM DTT and 0.23 mM PMSF) (Sigma Aldrich, St. Louis, MO). Each jejunum was then physically manipulated and tapped allowing the cells to separate from the interior surface. The jejunum was finally rinsed twice with 5 ml of EDTA buffer and all the fluid containing epithelial cells was collected, centrifuged at 300×g (Sorvell Rc5c) for 5 min, washed twice with 20 mL of balanced salt solution (BSS) containing 135 mM NaCl, 4.5 mM KCl, 5.6 mM glucose, 0.5 mM MgCl_2_, 10 mM HEPES and 1.0 mM CaCl_2_, pH 7.4, and the cells suspended in 2 mL of the same solution. Cell numbers were determined with hemocytometer and viABIlity (>90±5%) was assessed using trypan blue exclusion.

### Detection of β-Catenin Expression in Intestinal Cells by Immunoblot

Intestinal epithelial cells were isolated from the jejunum of AdRspo1- and AdLacZ-treated mice by modification of the protocol described by Weiser and Ferraris [27] as described in supplement. Isolated cells were fractionated as cytosolic and nuclear part by Nuclear/Cytosol Fractionation kit (Biovision Incorporated, Mountain View, California), according to the manufacturer's protocol and then subjected to immunoblot to analyze the β-catenin expression using mouse monoclonal antibody β-catenin (BD Bioscience, San Jose, CA). The immunoblot was developed and signal was detected by Chemiluminance assay (Amersham Pharmacia Biotech Inc, Piscataway, NJ). Purity of nuclear and cytosolic fractions was determined by the relative absence of β-tubulin and PCNA, respectively.

### RNA Isolation

Isolated murine intestinal epithelial cells were lysed using RLT buffer from RNeasy Mini Kit (Qiagen, Valencia, CA) and 1% betamercaptoethanol mix. Qiagen's protocol for the RNeasy Mini Kit with on-column DNA digestion was used to isolate RNA from the lysates. The RNA samples were stored at −80°C prior to use.

### Realtime PCR of β-Catenin Target Genes

To analyze the involvement of β-catenin downstream pathway in Rspo1 mediated intestinal repair mRNA levels of different β-catenin target genes in intestinal epithelial cells from from AdRspo1 and AdLacZ treated mice before and after WBI (10.4 Gy) were analyzed by real time PCR. cDNA was synthesized using the SuperScript™ First-Strand Synthesis System from Invitrogen. Realtime PCR was performed in Light Cycler real time PCR machine (Bio Rad Laboratories, Hercules, CA) using the ABsolute QPCR SYBER Green Mix (ABgene, Rochester, USA). The conditions followed the standard ABgene protocol with the exception for the annealing and extension step, where a temperature of 55°C for EphB2 and EphB3, 57°C for Tcf4, and 54°C for Lef1 were used for 30 seconds followed by 30 seconds at 72°C. To check for primer amplification specificity, a melting curve was generated at the end of the PCR and different samples containing the same primer pair showed matching amplicon melting temperatures. The gene sequences of β-catenin target genes were obtained from the Ensembl mouse genome database (http://www.ensembl.org/Mus_musculus/index.html) and the primers were designed using Primer3 software (http://frodo.wi.mit.edu/cgi-bin/primer3/primer3_www.cgi). Any primer pair generated with Primer3 was checked for gene specificity using the nucleotide-nucleotide BLAST database (http://130.14.29.110/BLAST/). The primer pairs used were as follows:

Beta actin: sense primer 5′ TGTACCCAGGCATTGCTGAC 3′ and anti-sense primer 5′ ACAGTGAGGCCAGGATGGAG 3′;

Ephb2: Sense primer 5′ AAGATGGGCCAGTACAAGGA 3′ and anti-sense primer 5′ CCAGCTAGAGTGACCCCAAC 3′;

Ephb3: sense primer 5′ TGGGACGGTACAAGGAGAAC 3′ and anti-sense primer 5′ TCATGTCCTGAATGCTGCTC 3′;

Tcf4: sense primer 5′ GGCGTTGGACAGATCACC 3′ and anti-sense primer 5′ GGTGAAGTGTTCATTGCTGTACTG 3′;

Lef1: sense primer 5′ AGACACCCTCCAGCTCCTGA 3′ and anti-sense primer 5′ CCTGAATCCACCCGTGATG 3′.

### Xylose Absorption Assay

To quantify intestinal absorption as a physiological indicator of mucosal barrier integrity in AdRspo1-, and AdLacZ-treated mice (n = 5/group) after WBI, a xylose uptake assay was performed, at various time points (1, 3.5, 7 and 10 days) after irradiation. A 5% w/v solution of D-xylose (100l/mouse) in deionized water was administered orally by feeding tube and 2 hrs post administration of D-xylose animals were sacrificed and blood samples collected using heparinized blood collection tubes (BD Biosciences, San Jose, CA). For determination of plasma D-xylose concentration a modified micromethod as reported by Eberts et al. was used [28]. One mL phloroglucinol (1,3,5-trihydroxybenzene, Sigma Chemical Co., St. Louis, MO) reagent (0.5 g of phloroglucinol, 100 mL glacial acetic acid and 100 mL of conc. HCL) was added to 10L of plasma. This solution was heated to 100°C in a water bath for 4 min to allow optimum color development. After equilibration to room temperature, sample absorption was determined with the aid of a spectrophotometer set at a wavelength of 554 nm.

### Kaplan-Meier Survival Curve Analysis

The effect of irradiation and concomitant Rspo1 on mice survival/mortality was analyzed by kaplan-Meier as a function of radiation (WBI and/or AIR) dose using Sigma–Plot and Graphpad Prism-4.0 software for Mac.

### Statistical Analysis of Digital Images

Sampling regions were chosen at random for digital acquisition for data quantitation. Digital image data was evaluated in a blinded fashion as to any treatment. A total of thirty to sixty crypts from two mice/treatment group were used for each data point. A two-sided student's t-test was used to determine significant differences between AdLacZ and AdRspo1 treated mice (P<0.05) with representative standard errors of the mean (SEM).
